# Microencapsulation of *Agave cupreata* Extract by Spray Drying: Physicochemical Properties and Antibacterial and Antiulcerogenic Activities

**DOI:** 10.1155/ijfo/9888736

**Published:** 2025-10-31

**Authors:** Cinthya Vanessa Calderón-Peralta, Ricardo Salazar, Liliana Alamilla-Beltrán, Mario Márquez-Lemus, Natividad Castro-Alarcón, Ma. Pilar Nicasio-Torres, Javier Jiménez-Hernández, Mónica Ramírez, Yaneth Castro-Coronel, Ma. Elena Moreno-Godínez, Patricia Álvarez-Fitz

**Affiliations:** ^1^ Maestria en Biociencias, Universidad Autónoma de Guerrero, Chilpancingo de los Bravo, Guerrero, Mexico, uagro.mx; ^2^ Investigadoras e Investigadores por México-SECIHTI, Universidad Autónoma de Guerrero, Chilpancingo de los Bravo, Guerrero, Mexico, uagro.mx; ^3^ Laboratorio de Microencapsulación, Escuela Nacional de Ciencias Biológicas-IPN, Ciudad de Mexico, Mexico; ^4^ Laboratorio de Biotecnología, Centro de Investigación Biomédica del Sur, Xochitepec, Morelos, Mexico

**Keywords:** *Agave cupreata*, antibacterial, antiulcerogenic, gum Arabic, isotherm adsorption, maltodextrin, spray drying

## Abstract

The *Agave cupreata* leaves are the main crop residues generated by the mezcal industry. It is known that agave leaves are potential sources of antibacterial and anti‐inflammatory compounds that could be used in the pharmaceutical industry. Therefore, the valorization of crop residues and maximal utilization of this material are of major research interest in the development of environmentally and sustainably produced products. In this study, the aqueous extract was microencapsulated (MCAC) from *Agave cupreata* leaves in order to evaluate its physicochemical properties, stability, and antibacterial and antiulcerogenic activity. The results showed that MCAC exhibited a spherical shape, concavities, and a rough surface. The phytochemical profile showed that MCAC presented flavonoids, terpenes, and saponins. Optimal storage conditions at 35°C for MCAC were determined from adsorption isotherms. The integrity of MCAC was observed up to a water activity of 0.436. The results of the antibacterial activity demonstrated a growth inhibitory effect of PAC and MCAC on Gram‐negative bacteria at a concentration of 32 mg mL^−1^. In animal experiments, compared with the negative control (absolute ethanol), MCAC and PAC powders exerted a protective effect against ethanol‐induced gastric ulcers, with protection rates of 34.45% and 92.24%, respectively. The results suggest that the powder obtained in the present study may be useful as a food additive and/or as an ingredient of pharmaceutical drugs.

## 1. Introduction

The genus *Agave* has 210 species, among which 159 are found in Mexico (119 endemic); thus, Mexico is considered a center of *Agave* biodiversity [1]. Although the main use for *Agave* is to produce alcoholic beverages such as tequila, mezcal, and *bacanora*, there is a growing interest in the potential pharmacological use of *Agave* extracts. Several studies have shown that the extracts obtained from different parts of A*gave* plants exhibit antibacterial [2], anti‐inflammatory [3], antioxidant [4], and antitumor activities [5]. It has been demonstrated that the extracts obtained from *Agave* plants contain phenols, flavonoids, sterols, fructanes, steroidal saponins, and terpenes [2, 6]. However, these data were obtained mainly from nonpolar or medium‐polarity extracts, minimizing reports on aqueous extracts (AEs), in part because the water extract stability may be affected by environmental factors such as exposure to daylight, pH, temperature, and moisture [7]. One way to increase the stability and viability of AE is through microencapsulation, the process by which these natural bioactive compounds are encapsulated to protect them from degradation under various processing and storage conditions. During this process, the microsized particles are covered by wall material/encapsulant/shell material, which protects and isolates these particles from ambient conditions [8].

One of the techniques to generate microcapsules or microparticles is the spray‐drying (SD) process. This is a popular and well‐established method; the principle of encapsulation by SD is related to its ability to enclose active components within a protective outer layer, while the liquid feed is transformed into a dry, stable form [9]. The advantages of this technique include reduced production times and that the drying process is performed without the need for high temperatures, allowing the retention of the compound’s properties such as color, flavor, and chemical composition [10]. Several encapsulating agents can be employed for microcapsule production by SD. Some of the characteristics that may be considered when choosing encapsulating agents include their film‐forming ability, biodegradability, resistance to acidic pH, viscosity, solids content, hygroscopy, and solubility (S) [11].

In Guerrero State, Mexico, *Agave cupreata* (*A. cupreata*) Trel. & A. Berger, commonly known as *Agave papalote*, is an endemic species and represents the raw material for producing mezcal, a traditional alcoholic beverage. Additionally, these plants have been employed in traditional medicine to treat conditions such as pain and to act as antioxidant, anti‐inflammatory, and antimicrobial agents, among others [12].

*Agave* stem and leaf bases, commonly referred to as “head or *piña*,” are the commercially relevant parts of the plant for mezcal production. The leaves represent 46% of the total plant weight. They are disposed of during mezcal production and are considered agroindustrial waste [13]. There are a few studies on the phytochemical and biological activity of extracts from *A. cupreata* leaves: Salazar‐Pineda et al. [3] evaluated the antibacterial and anti‐inflammatory activities of leaf extracts (hexane, dichloromethane, and acetone). These authors found that the extracts inhibited the growth of Gram‐negative and Gram‐positive bacteria (minimal inhibitory concentration (MIC), 2–16 mg mL^−1^). The authors also found that the extracts exhibited anti‐inflammatory activity in murine models; the dichloromethane and acetone extracts presented an inhibitory effect on the formation of edemas of 62.47%–64.29% and 40.82%–48.82%, respectively.

The preliminary phytochemical profile of the *A. cupreata* leaf extract revealed the presence of anthrones, anthraquinones, coumarins, alkaloids, essential oils, saponins (derived from chlorogenin and thiogenin), and pungent compounds [3, 14]. Regarding the AE of this plant, there are, to our knowledge, no reports of its phytochemical profile or biological activity; therefore, the objective of this study was to microencapsulate the AE from *A. cupreata* leaves and to evaluate their physicochemical properties, stability, and antibacterial and antiulcerogenic activities.

## 2. Materials

### 2.1. Reagents

The reagents used in this study included gum Arabic (GA) E_414_ (Morevo Quick Gum); maltodextrin (MD) DE_20_ (Globe Corn Product International, Illinois, United States); silica gel 7733 (Merck KGaA, Darmstadt, Germany); LiCl, KC_2_H_3_O, MgCl_2_, K_2_CO_3_, Mg (NO_3_)_2_, NaNO_3_, NaCl, and KCl (Sigma‐Aldrich, Merck); amikacin (Laboratorios PiSA, S.A de C.V); Thiazolyl Blue Tetrazolium Bromide (MTT) (Sigma‐Aldrich, Merck); silica gel 7733 (Sigma‐Aldrich, Merck); and absolute ethanol (Merck KGaA, Darmstadt, Germany).

### 2.2. Vegetal Material

Fresh leaves (5 kg) of *A. cupreata* were collected in Totomochapa, Municipality of Tlapa de Comonfort in Guerrero State, Mexico (longitude: −98.459167; latitude: 17.541111). The plant was identified by Dr. Abisaí Josué García‐Mendoza of the Instituto de Biología of the Universidad Nacional Autónoma de México (UNAM), Mexico. A voucher specimen with accession number MEXU‐2050 was submitted to the National Herbarium of Mexico (MEXU) and was identified as *A. cupreata* Trel. & A. Berger.

### 2.3. AE Preparation and the SD Process

For AE preparation, fresh leaves from *A. cupreata* were processed by the infusion technique described by Mena et al. [15]. Briefly, 1700 g of leaves was subjected to infusion in distilled water (8 L) at 60°C for 2 h. The AE was filtered through Whatman No. 4 filter paper (25‐*μ*m pore). The AE obtained had 1.6° Brix and total solids of 21 g/L.

The microcapsules of AE of *A. cupreata* (MCACs) were obtained by the SD method. Briefly, these were prepared by mixing 100 mL of AE with GA and MD at a proportion of 20:80 *w*/*w*. Then, the mixture was homogenized at 6098 g during 5 min in an ULTRA‐TURRAX (IKA, Germany) at room temperature (25°C). The SD of the samples was carried out in a spray‐dryer (Mobile Minor; GEA Niro, Denmark) employing a pneumatic nozzle as atomizer, with inlet‐air and outlet‐air temperatures of 180°C and 80°C, respectively [16].

Additionally, AEs were processed under the conditions described previously, without the addition of encapsulating agents to obtain AE powder (PAC). Both MCAC and PAC were collected in hermetically sealed aluminum Ziplock bags, stored in a glass flask containing silica gel 7733, and were protected from light at room temperature.

### 2.4. Physicochemical Analysis

#### 2.4.1. Drying Yield (DY)

The solids content of the MCAC and PAC was determined prior to their undergoing SD, using a refractometer (HI96814; Hanna, United States). The DY was calculated by the ratio between the powder product collected at the end of the SD and the content of solids in the AE and MCAC mixture utilized initially to feed the SD system. The DY was calculated using the following equation:

(1) %DY=total collected powder on dry basis gtotal solids content in feed g∗100.

#### 2.4.2. Moisture Content (MC) and Water Activity

MC was determined according to the AOAC method (AOAC 930.15, 2015). The MCAC and PAC (1 g) were placed on aluminum trays and dried at 105°C/1 h in a drying oven (Grieve LR‐271C; United States). To calculate the MC of each sample, the following equation was utilized:

(2) %MC=sample wet weight g−sample dry weight gsample wet weight g∗100.

The water activity (*a*_*w*_) of the MCAC and PAC was calculated by direct reading in an electronic system (Aqualab Dew Point 4TEV) after stabilizing the samples at 25°C.

#### 2.4.3. Solubility

Solubility (S) was determined according to the method proposed by Rezende et al. [17]. The MCAC or PAC (1 g) was mixed and homogenized with 100 mL of distilled water in a magnetic stirrer (S131125q, CIMAR EC, México) for 30 min. Subsequently, the solution was centrifuged (Eppendorf 5810R) at 3000 g for 5 min. A total of 25 mL aliquots of the supernatant were taken, transferred onto previously weighed glass Petri dishes (Corning, Germany), and immediately placed in a drying oven at 105°C/5 h. To calculate the S of each sample, the following equation was used:

(3) %S=final sample weight ginitial sample weight g∗100.

#### 2.4.4. Color Parameters

Color analysis of the MCAC and PAC was carried out with a Ci62 X‐Rite Spectrocolorimeter (X‐Rite, Grandville, Michigan, United States) following the CIELAB system (*L* ^∗^, *a* ^∗^, and *b* ^∗^), where the value of *L* ^∗^ indicates brightness (0 = black and 100 = white), *a* ^∗^ = (−*a* ^∗^ [green]/+*a* ^∗^ [red]), and *b* ^∗^ = (−*b* ^∗^ [blue]/+*b* ^∗^ [yellow]). The value of chroma ($C^{*}=\sqrt{{b^{*}}^{2}+{a^{*}}^{2}}$) indicates the purity or saturation of the color. The Hue angle (*H* ^0^ = tan − 1*b* ^∗^/*a* ^∗^) indicates the color of the sample (i.e., 0°–360° = red, 90° = yellow, 180° = green, and 270° = blue). Negative values of *H* ^0^ were converted into positive values by adding 180° to fit into the 90°–180° quadrant [18].

#### 2.4.5. Physical Stability of the Powders

The MCAC and PAC ( ~ 500 mg) were placed on aluminum supports inside the glass adsorption cells with saturated solutions of LiCl, KC_2_H_3_O, MgCl_2_, K_2_CO_3_, Mg (NO_3_)_2_, NaNO_3_, NaCl, and KCl to provide different *a*_*w*_ 0.108, 0.215, 0.318, 0.436, 0.515, 0.628, 0.743, and 0.821, respectively [19]. The adsorption cells were placed at 35°C and weighed every 5 days for 20 days (when the difference between two consecutive weights was less than 0.005 g). The Guggenheim–Anderson–de Boer (GAB) equation was employed to model the water adsorption isotherms, calculated using the following equation:

(4) M=M0C kaw1−kaw 1−kaw+Ckaw,

where *a*_*w*_ is the water activity; *M* is the water content of the sample on a dry basis; *M*_0_ is the monolayer water content; *C* is the Guggenheim constant, given by *C* = *c* ^′^exp (*h*_*m*_ − *h*_*n*_)/RT, where *c* ^′^ is the constant equation, *h*_*m*_ is the heat of sorption of the first layer, *h*_*n*_ is the heat of sorption of the multilayer, *R* is the gas constant, and *T* is the absolute temperature; and *k* is the constant correction property of multilayer molecules with respect to bulk liquid, given by *k* = *k* ^′^exp (*h*_1_ − *h*_*n*_)/RT, where *k* ^′^ is the constant of the equation and *h*_1_ is the heat of the condensation of pure water.

The values of the parameters of the GAB equation (*M*_0_, *C*, and *k*) were estimated by adjusting the mathematical model to the experimental data, using a nonlinear regression with Kaleidagraph Version 4.0 software (Synergy Software, Perkiomen, United States). Accuracy of fit was evaluated using the average in the relative percentage difference between the experimental and the predicted values of the MC or the mean relative deviation (*P*) module defined by the following equation:

(5) P%=100N∑i=1NMei−MciMei,

where Me_*i*_ is the MC in the observation, Mc_*i*_ is the MC predicted in that observation, and *N* is the number of observations. In general, a good fit is obtained when *p* < 10*%* [20].

##### 2.4.5.1. Micropore Volume

The MC corresponding to the micropore volume was determined from the data generated with the adsorption isotherms, adjusting the Dubinin–Radushkevich model and utilizing the following equation [21]:

(6) logn=log n0−B log2Pv0PV,

where *n* is the MC adsorbed on a dry basis, *n*_0_ is the amount of water adsorbed corresponding to the micropore volume, and *B* is a constant related to the adsorbent structure that indicates the dispersion of the pore‐size distribution [22].

#### 2.4.6. Morphology

The morphology of the MCAC and PAC was visualized by scanning electron microscopy (SEM) in an environmental microscope (Philips Model XL30, ESEM) with a 10–20 kV beam and a gaseous secondary electron detector. The samples were mounted on stainless steel sheets and coated with graphite utilizing a JFC‐1100 Sputter Coater (JEOL, Akishima, Japan) before analysis.

The images were taken at different magnifications (1000×, 2000×, and 2500×). Average particle size was calculated with the ImageJ Version 1.57 software program (NIH, United States) from the images obtained from SEM.

#### 2.4.7. Qualitative Phytochemical Profile

Thin‐layer chromatography (TLC) was carried out according to that described by Salazar‐Pineda et al. [3]. The MCAC and PAC were dissolved in water and spotted on silica gel 60 RP‐18 TLC plates (Merck KGaA, Darmstadt, Germany); the plates were developed in the ethanol:acetone solvent system (7:3 *v*/*v*). After being removed from the developing chamber, the plates were air‐dried in a fume hood. For identification of the phytochemical profile, TLC plates were visualized under UV light at a 254‐nm wavelength and revealed with chromogenic developers [23].

### 2.5. Antibacterial Activity

#### 2.5.1. Microorganism Strains

The following six strains were obtained from the American Type Culture Collection (ATCC): *Enterobacter cloacae* 700323, *Escherichia coli* 25923 and 35218, *Salmonella Dublin* 9676, *Staphylococcus aureus* 25923, and *Enterococcus faecalis* 29212. Clinical isolates of *Klebsiella pneumoniae*, *Escherichia coli*, *Staphylococcus hominis*, *Staphylococcus haemolyticus*, and *Staphylococcus aureus* were from the Laboratorio de Investigación Microbiológica strain collection. Bacterial inoculum was obtained according to the standard 0.5 of the McFarland scale.

#### 2.5.2. Minimum Inhibitory Concentration

The MIC of the samples was determined by the microtiter broth dilution method [3]. The MCAC and PAC were evaluated at concentrations ranging from 1 to 32 mg mL^−1^. Amikacin (100 *μ*g mL^−1^) was used as a positive control. The MIC was defined as the lowest concentration at which the MCAC and PAC inhibit bacterial growth; this was visually determined as a color shift of the MTT from yellow to purple [24].

### 2.6. Antiulcerogenic Activity

Female albino ICR mice (weighing 25–30 g each) were obtained from the animal facility of Universidad Autónoma del Estado de Morelos (Morelos State, Mexico). The animals were maintained under standard laboratory conditions (25°C, 12‐h light/12‐h dark, and water/food ad libitum) and were acclimatized for 1 week prior to the experiments. All determinations were carried out employing the minimal number of animals, following the protocol of the Institutional Research Committee of the Mexican Social Security Institute (R‐2011‐1701‐3) and Official Mexican Standard NOM‐062‐ZOO‐1999. Prior to the induction of the gastric ulcer, the animals were fasted for 24 h to ensure an empty stomach and were individually housed in cages with wire mesh at the bottom to prevent coprophagia.

#### 2.6.1. Induction of Gastric Ulcer With Ethanol

Sixteen mice were assigned to four groups of four mice each. The animals in Group I received no treatment during the experimental period and served as a normal control, while animals in Group II (negative control) were administered water. However, animals in Groups III and IV (experimental controls) were administered 100 mg kg^−1^ body weight (BW) of MCAC and PAC (per os) dissolved in water, respectively.

After 1 h of treatment, the negative and experimental controls received 250 *μ*L per os of absolute ethanol (10% of BW) for the induction of gastric ulcer. One hour after ethanol administration, the animals were anesthetized by an intraperitoneal injection of sodium pentobarbital (10% of BW) and then sacrificed by cervical dislocation, their stomachs were rapidly removed, and they were opened along the greater curvature and rinsed with water to remove gastric contents and blood clots [25]. The stomach was photographed with a digital camera (Nikon, 200 MPX) and analyzed using image analysis software (ImageJ).

The total surface area of each stomach (mm^2^) was determined, as well as the number and severity of the hemorrhagic lesions per stomach, and these were expressed as total ulcerated gastric area (mm^2^). The means of each group were calculated and expressed as the ulceration index (UI) percentage and the protection percentage [26]. The formula for the UI and the protection percentage was calculated using the following equations:

(7) UI %=ulcerated areatotal stomach area∗100,

(8) Protection %=UI control−UI‐treated groupUI control∗100.

### 2.7. Statistical Analysis

Data were expressed as the mean ± the standard deviation. Each determination was carried out in triplicate. A one‐way analysis of variance (ANOVA) followed by a Student *t*‐test or Dunnett test was performed using the SigmaPlot Version 11.0 statistical software program. A significant statistical difference was considered with values of *p* < 0.05.

## 3. Results and Discussion

### 3.1. Physicochemical Properties

#### 3.1.1. Drying Yield

The DY in SD encapsulation refers to the proportion of the starting material that is converted into a dry powder product. It is a crucial parameter for assessing the efficiency of the encapsulation process. The DY for the MCAC (using GA and MD) was 82.90% in comparison with that of the PAC (43.96%) (Table 1). This increase in the MCAC DY can be attributed to the addition of wall material (GA and MD), and several studies showed that the use of solely MD resulted in yields between 44.7% and 66.7% [27].

**Table 1:** Physicochemical properties and estimated GAB parameters of MCAC and PAC.

**Parameters**	**MCAC**	**PAC**
Drying yield (%)	82.90	43.96
Moisture content (%)	2.82 ± 0.104	4.72 ± 0.284^∗∗^
*a*_*w*_	0.247 ± 0.019	0.278 ± 0.013
Solubility (%)	25.57 ± 1.22^∗∗^	17.50 ± 1.13
Color:		
*L* ^∗^ value	38.64 ± 1.2^∗^	36.46 ± 0.2
*a* ^∗^ value	−0.84 ± 0.02	−0.88 ± 0.03
*b* ^∗^ value	4.80 ± 0.09	7.26 ± 0.24^∗∗^
Chroma	4.72 ± 0.09	7.2 ± 0.25^∗∗^
*H* ^0^	91.18 ± 0.43^∗∗^	87.69 ± 0.56
*M*_0_	7.95	13.28
*C*	1.98	1.29
*k*	0.88	0.85
*%* *P*	7.59	3.47
*N*	5.91	2.96

*Note:* *L*: brightness; *b* ^*^: −*b* (blue), +*b* (yellow); *a* ^*^: −*a* (green), +*a* (red); *M*_0_: the moisture content in the monolayer (g water/100 g dry solid); *C*: constant; *k*: constant; *%* *P*: setting value; *N*: micropore volume (g water/100 g dry solid). Student’s *t*‐test.

Abbreviations: *a*_*w*_: water activity; MCAC: *A. cupreata* powders GA + MD (20:80 *w*/*w*); PAC: *A. cupreata* AE powders.

*p < 0.01.

**p < 0.001.

Additionally, Caliskan and Dirim [28] reported that increasing the concentration of the wall material favors DYs, since when using small amounts of MD, the feed liquid may adhere to the drying chamber because of the sticky behavior of the extracts with high sugar content. However, using higher amounts of wall materials increases the glass transition temperature and thus reduces this sticky behavior.

Also, the addition of GA (low viscosity and high S) increases the DY in that the GA is a good emulsifying material, facilitating an efficient entrapment of the components exhibiting film‐forming and plastic behavior rather than a glassy property, which prevents the cracking of the protection matrix [29].

On the other hand, the lower DY in the PAC (43.96%) may be due to that it does not contain wall material, increasing its adherence in the dryer chamber as well as to cyclones, causing low efficiency in the collection of powders [30]. In this sense, Muzaffar et al. [31] reported that the adherence of the drops could be related to the content of the sugars and organic acids present in the samples, which would give rise to a problem of powder stickiness. In this regard, several studies report that *Agave* AEs contain sugars and fructooligosacharides (FOSs), and this carbohydrate can significantly impact powder stickiness, especially during the SD process, due to its hygroscopic nature and its low glass transition temperature [32, 33].

#### 3.1.2. Moisture Content and Water Activity

Moisture content (MC) and water activity (*a*_*w*_) are important parameters indicating the quality and storage stability of microencapsulates. The MC influences the physicochemical, microbial stability (MC content in the 1%–6% range is sought by industry to ensure the stability of powder during storage), whereas the *a*_*w*_ is useful for determining nonenzymatic and enzymatic activities, microbial growth, and lipid oxidation in foods [34].

The MCAC exhibited lower MC (2.82%) compared to PAC (4.72%) (*p* 0.001). This decrease may be due to the addition of GA and MD; Gabas et al. [35] reported that the addition of GA and MD to pineapple pulp powders results in decreased MC, and the authors suggest that this could be due to the reduction in the number of exposed polar groups, which results in lower water retention after drying; on other hand, Castañón‐Rodríguez et al. [36] suggested that MD facilitates the diffusion of water molecules in the vapor state toward the outer side of the particles. In contrast, the PAC exhibits higher MC, and this effect could be due to the presence of sugar; in this context, Shrestha et al. [37] reported that the components that possess sugars in their structure are highly hygroscopic and easily absorb moisture from the air.

Water activity (*a*_*w*_) is one of the important parameters that affect the shelf life and quality of microcapsules, as well as their participation in chemical and biochemical reactions [38]. The results obtained showed no significant difference in *a*_*w*_ between MCAC and PAC (*p* >  0.05) (Table 1). However, both MCAC and PAC have an *a*_*w*_ below 0.3, and according to Bicudo et al. [39], powders with an *a*_*w*_ below 0.3 are considered microbiologically and chemically safe, since a higher *a*_*w*_ would favor bacterial and fungal growth [34].

#### 3.1.3. S

The structural and functional properties of wall materials are contributors to their functional features, such as S. S is an important parameter to determine the particle‐dissolution stage. In this study, it was found that the GA/MD ratio in the wall materials improved S regarding MCAC in comparison with the PAC (*p* < 0.001) (Table 1). This phenomenon has been attributed to the addition of wall material (GA and MD) to the AE, which may result in changes in the interactions between the water molecules and the chemical compounds in the solution [40], which in turn may reduce the number of polar groups exposed, favoring the formation of smaller particles with increased surface area during the SD process [35]. Also, Goula and Adamopoulos [41] report that MD is one carrier that is mainly used in the SD process due to its physical properties, including high S in water; hence, S powders increase as the amount of MD increases.

#### 3.1.4. Color Parameters

Chroma values and CIELAB color coordinates are presented in Table 1. As expected, the encapsulates obtained with encapsulating material (MCAC) presented high *H* ^0^ (closest to the angle for yellow), *L* ^∗^ luminosity, and low chroma (*C* ^∗^) in comparison with the PAC (*p* = 0.001).

For the parameter of *a* ^∗^, both powders present negative values, indicating a tendency toward a greenish color, and in positive values for *b* ^∗^ (in the case of the PAC, higher values of *b* ^∗^ were observed), denoting a yellow coloration. The results obtained from the *a* ^∗^ and *b* ^∗^ values reveal that the data fall in the first quadrant (−*a*, +*b*), indicating a green and yellow trend. This result was expected, in that the color of AE from *A. cupreata* is yellowish‐green.

Hue angle (*H* ^0^) and chroma (*C* ^∗^) comprise important color attributes that characterize the perception, the intensity, and the purity of the color [42], respectively. The results obtained showed that the powders presented high *H* ^0^ values (closest to the angle for yellow), particularly the MCAC in comparison with the PAC (*p* = 0.001). The powders exhibited low values of the chroma parameter, indicating the low intensity of the color. However, the MCAC demonstrated a low chroma in comparison with the PAC (*p* < 0.001); this phenomenon could be due to the wall materials used, because MD has been reported to mask the colors when used for the obtention of powders [43].

#### 3.1.5. Physical Stability of the Powders

To determine the stability of the MCAC and PAC under different conditions of humidity, water vapor adsorption isotherms at 35°C were performed (Figure 1). The MCAC and PAC exhibited Type III (nonsigmoidal) adsorption isotherms (Figure 1a). Nonsigmoidal isotherms are characterized by having a lower water adsorption capacity at low water activity values [44]. The adsorption isotherms obtained in this study were different from those reported by Acosta‐Domínguez et al. [45], who obtained a Type II isotherm for fructans extracted from *Agave tequilana.* The isotherm of PAC at 35°C exhibited a higher water adsorption capacity than MCAC. This behavior was attributed to the amount of exposed polar groups present in PAC.

Figure 1Adsorption isotherms of MCAC and PAC at 35°C. (a) MCAC: *A. cupreata* powder GA + MD (20:80 *w*/*w*, open square) and PAC: *A. cupreata* EA powders (filled circle). The continuous line represents the fitted points using the GAB model. (b) Representative images of MCAC and PAC under different water‐activity conditions at 35°C.

**Figure:**
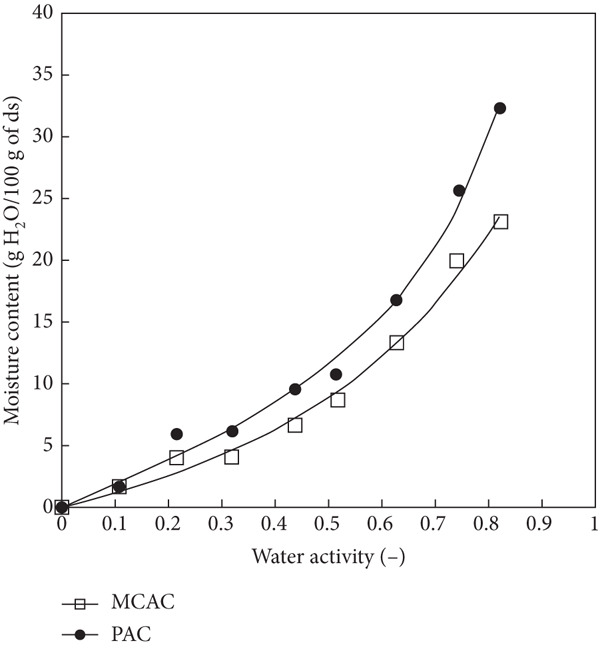
(a)

**Figure:**
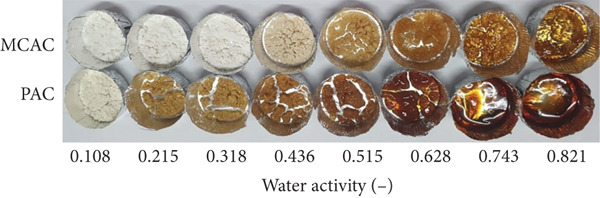
(b)

The parameters *M*_0_, *C*, and *k* calculated by the GAB model, as well as the MC corresponding to micropore volume (*N*), are presented in Table 1. The *M*_0_ value of the MCAC was 1.6 times lower than that of the PAC. The values of *C* and *k* were similar for both analyzed powders. It is noteworthy that, despite that the GAB model demonstrates a good fit with respect to the experimental data with a *%* *P* value of 3.4%–7.5%, the calculated *M*_0_ values were overestimated. The *a*_*w*_ at 35°C corresponding to *M*_0_ predicted by the GAB equation was 0.47 and 0.55 for MCAC and PAC, respectively. As can be observed in Figure 1b, at those *a*_*w*_ values, both MCAC and PAC powders are completely solubilized. In contrast, studies have demonstrated that *N* provides a more realistic monolayer value [22]. The *a*_*w*_ at 35°C corresponding to *N* was 0.39 and 0.18 for MCAC and PAC, respectively. As can be observed in Figure 1b, at those *a*_*w*_ values, both MCAC and PAC powders maintain their physical stability; thus, the micropore volume (*N*) is a useful parameter for anticipating the appropriate storage conditions of MCAC and PAC powders in relation to ambient relative humidity. By increasing the *a*_*w*_ to above the maximal stability values, a change in the physical characteristics of the MCAC and PAC was observed; this type of behavior is typical of sugar‐rich systems and is probably due to the gradual dissolution of sugar, which results in a complete leaching of sugar in solution [46].

#### 3.1.6. Morphology

**Figure 2:**
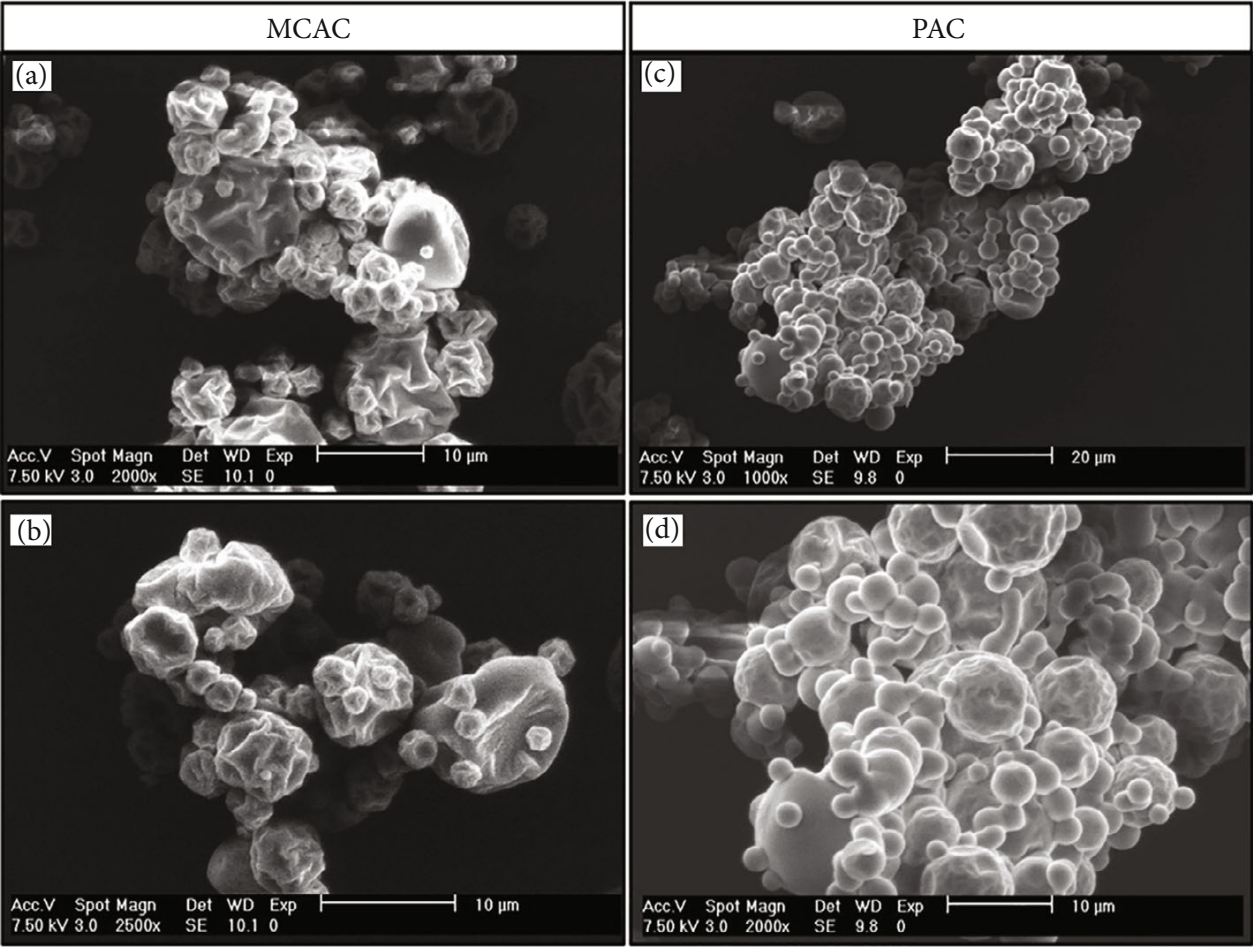
Scanning electron microscopy (SEM) images of *A. cupreata* powders. MCAC: *A. cupreata* microcapsules GA + MD (20:80 *w*/*w*) ((a) (2000×) and (b) (2500×)). PAC: *A. cupreata* AE powder ((c) (500×) and (d) (1000×)).

The surface morphology of the powders was observed; the representative images of the MCAC and PAC are presented in Figure 2. The micrographs revealed that the MCAC exhibited an irregular spherical shape with a rough surface (Figure 2a,b), whereas the PAC exhibited a spherical shape with slight roughness (Figure 2c,d). This phenomenon could be attributed to the contraction of sprayed droplets as the solvent is lost by rapid evaporation; within the droplet, the solute molecules organize according to the diffusion rates. During this stage, contractions occur, solidifying the external part of the particle, which slows the transport of the solvent to the surface for evaporation [17].

The use of wall materials during the SD process exerts a significant effect on the behavior of the molecules in the droplet, thus on the final morphology of the dried particles [29]. Several authors have reported the effect of wall material: In this context, Gallegos‐Infante et al. [47] reported that the addition of GA increases roughness and prevents the formation of wall cracking, mainly due to the plasticizing properties of the GA, while the use of low equivalents of MD results in dented, irregular, rounded spheres without ruptures, and Lopera et al. [48] mentioned that the combination of GA and MD gives rise to an increase in the number of folds on the surface of the particles.

Another of the important factors that contribute to morphology is the relationship among the wall materials. This was observed by Tolun et al. [29] in the microencapsulation of grape polyphenols when different formulations were carried out with MD and GA, with the authors reaching the conclusion that the 80:20 MD:GA relationship afforded these authors better results in terms of morphology. The latter was employed in this study, where we obtained powders with a slightly rough surface with some smooth sections; according to the literature, the preferred structure in microcapsules usually exhibits a spherical shape, with a low average size ranging from 5 to 50 *μ*m, with a smooth outer surface or one exhibiting the formation of teeth or concavities with an irregular shape [49].

#### 3.1.7. Qualitative Phytochemical Profile

Knowing the phytochemical profile of encapsulated products is crucial, as it allows us to determine the active ingredients present in the encapsulated products and whether the encapsulation process exerts any degrading effects on them.

The qualitative phytochemical profile of the MCAC and PAC revealed the presence of flavonoids, terpenes, and saponins. Although there are, to our knowledge, no other reports on the MCAC and PAC from *A. cupreata*, Urbina et al. [14] and García‐Morales et al. [50] identified saponins derived from tigogenin and chlorogenin from the methanolic extract of *A. cupreata* leaves. Moreover, several studies have identified similar compounds in polar extracts obtained from the leaves of different species of *Agave* [6, 51]. We found that the MCAC and PAC contain saponins and terpenoids, but not tannins or anthocyanins. These data are in agreement with other reports in which no tannins or anthocyanins were detected in the EA of *Agave* leaves, but these, nonetheless, were found in hydroalcoholic extracts [52].

### 3.2. Antibacterial Activity

To evaluate the antibacterial activity of the MCAC and PAC, we calculated the MIC (Table 2). We observed that PAC and MCAC exhibited antibacterial activity against Gram‐negative strains of *Escherichia coli* and *Salmonella Dublin*, with MIC values of 16 and 32 mg mL^−1^. However, we observed that this activity decreased on adding wall materials. The PAC demonstrated the lowest MIC 16 mg mL^−1^ against ATCC *Escherichia coli* (25923 and 35218), *E. coli* clinical isolate, and *Salmonella Dublin* (9676), which was two times less than the MIC of the MCAC (32 mg mL^−1^) for the same bacteria. The loss of biological antibacterial activity by the addition of wall material has been reported, and this effect could be due to the addition of wall materials reducing or eliminating the antibacterial activity, because wall material acts as a barrier, preventing or hindering the interaction of the encapsulated with the bacteria [53].

**Table 2:** Minimal inhibitory concentration (MIC mg mL^−1^) of MCAC and PAC.

**Bacterial strains**	**MCAC**	**PAC**	**Negative control**	**Positive control**
ATCC				
*E. cloacae* 700323	>64	>64	+	−
*E. coli* 25923	32	16	+	−
*E. coli* 35218	32	16	+	−
*S. Dublin* 9676	32	16	+	−
*S. aureus* 25923	>64	>64	+	−
*E. faecalis* 29212	>64	>64	+	−
Clinical isolates				
*K. pneumoniae*	>64	>64	+	−
*E. coli*	32	16	+	−
*S. hominis*	>64	>64	+	−
*S. haemolitycus*	>64	>64	+	−
*S. aureus*	>64	>64	+	−

*Note:* Negative control: +, with growth. Positive control: antibiotic amikacin (100 *μ*g mL^−1^); −, no growth.

Abbreviations: MCAC: *A. cupreata* powders GA + MD (20:80 *w*/*w*); PAC: *A. cupreata* AE powders.

Several studies demonstrated that extracts from different species of *Agave* exhibit antibacterial activities [3, 6]. It has been shown that medium‐polarity extracts obtained from the leaves of *A. cupreata* possess antibacterial activity, mainly against Gram‐positive strains [3]. In this work, we found that the MCAC and PAC obtained from an EA of *A. cupreata* leaves revealed antibacterial activity against Gram‐negative ATCC strains (*E. coli* 25923, *E. coli* 35218, and *S. Dublin* 9676), as well as a multiresistant clinical isolate (*E. coli*) with an MIC of 16–32 mg mL^−1^. There are few studies on the antibacterial activity of *A. cupreata* extracts. However, it has been shown that polar extracts from other species of *Agave*, such as *A. sisalana*, *A. intermixta*, and *A. attenuata*, demonstrated an inhibitory effect on Gram‐negative bacteria, such as *E. coli*, *Shigella dysenteriae*, *Salmonella typhi*, and *E. faecalis*, with MIC values of 10–140 mg mL^−1^ [52].

The differences among the antibacterial activity from extracts obtained from different *Agave* species may be rendered by the type of secondary metabolites contained in the extract [6]; in this regard, we found that MCAC and PAC contain flavonoids, terpenoids, and saponins, the latter already reported by Urbina et al. [14]. It has been reported that these compounds possess antimicrobial effects because they could alter the membrane properties of bacteria, such as hydrophobicity, surface charge, and membrane integrity, which are followed by a release of intracellular compounds and subsequently, cell death [4].

### 3.3. Antiulcerogenic Activity

**Figure 3:**
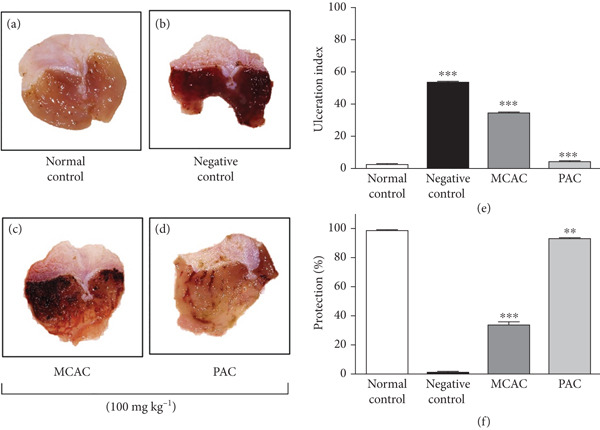
Effect of MCAC: *A. cupreata* microencapsulated GA+MD (20:80 *w*/*w*) and PAC: *A. cupreata* AE microparticles, the protection percent and ulceration index in the ethanol‐induced gastric ulcer model (*n* = 4). (a) Normal group, (b) negative control (water), (c) MCAC (100 mg kg^−1^ body weight), (d) PAC (100 mg kg^−1^ body weight), and (e) ulceration index on the ulcer gastric. (f) Protection effects on ulcer gastric. Each value represents the mean ± SD of four animals. One‐way analysis of variance (ANOVA) followed by a post‐Dunnett test. Differences from negative control.  ^∗∗∗^ *p* = 0.0001 and  ^∗∗^ *p* = 0.001.

The antiulcerogenic effect of the MCAC and PAC was evaluated in a murine model of ethanol‐induced gastric ulcer. The macroscopic appearance of mouse stomachs with ethanol‐induced gastric ulcer is presented in Figures 3a, 3b, 3c, and 3d. As shown in Figure 3, stomachs from normal control mice (Figure 3a) exhibited a homogeneous pinkish color, whereas stomachs from negative control mice (Figure 3b) presented large areas of erythema (red tissue) and linear lesions (hemorrhages/ulcers) indicative of mucosal damage. However, pretreatment with MCAC and PAC demonstrated a significant reduction in erythema and ulceration (Figure 3c,d).

The treatment with either PAC or MCAC (100 mg kg^−1^ BW) exerts a gastroprotective effect against gastric ulceration, promoting 92.24% and 34.45% of protection (*p* = 0.0001) and exhibited an UI of 4.05% of PAC and 34.45% of MCA, as compared to the negative control, revealing that the addition of wall material decreased the biological activity (Figure 3e,f).

This inhibitory effect of the PAC and MCAC may be due to the presence of the saponins, terpenoids, and flavonoids reported in the phytochemical profile. Different studies have related this activity to the steroidal saponins [54], in that these latter possess the ability to increase the mucosa‐defense factors, inducing the renewal of glycoproteins, therefore increasing the amount of cell mucus, in this manner preventing the necrotizing agent (ethanol) from penetrating or interacting with the macromolecules of the gastric mucosa [55].

Additionally, studies in animal models of induced ulcer report that some metabolites of fruits and vegetables demonstrate protective activity by raising the levels of prostaglandins (PGE2) and glycogen, as well as by decreasing the pepsin [56]. Also, it has been reported that secondary metabolites (flavonoids, terpenes, terpenoids, saponins, phenolic acids, tannins, and fatty acids) possess gastroprotective effects and promote the secretion of gastric mucus [57]. On the other hand, there are very few studies, to our knowledge, that show the anti‐inflammatory activity of *A. cupreata* [3], and there are no studies, to our knowledge, on its gastroprotection. However, the genus *Agave* exhibited antiulcerogenic activity; for example, El‐Hawary et al. [58] reported that *Agave angustifolia* var. Marginata possessed the highest ulceroprotective activity, which could be attributable to the high abundance of various saponins and homoisoflavonoids. Also, Pereira et al. [54] reported that furostanol and spirostanol steroidal saponins isolated from *A. angustifolia* var. Marginata exert a gastroprotective effect of 60%–70% at the administered concentrations of 100 mg kg^−1^ per os.

## 4. Conclusions

The results show that SD with the use of GA and MD as wall materials (20:80 *w*/*w*) comprises a suitable method to obtain encapsulates of AE from *A. cupreata*. The use of wall material does not affect the profile of the bioactive compounds (flavonoids, terpenes, and saponins), but it does have an effect on increasing stability in encapsulates, while modifying certain properties, such as particle size, MC, color, and S, parameters that afford them advantages if they were to be used as drugs or functional foods.

When evaluating the biological activities (antibacterial and antiulcerative), it was determined that the addition of the wall materials does not increase the effect; this suggests that there may be certain limitations in the encapsulated extracts used of wall material such as GA and MD.

However, PAC and MCAC showed promise for use as functional food or pharmaceutical products due to their color, chemical profile, physicochemical properties (moisture, water activity, and morphology), and biological activities, this being an attractive prospect for these markets. Further studies are required in order to better obtain insight into the effects of the addition of other wall materials and other biological activities (in vitro and in vivo).

## Ethics Statement

Studies on animals were conducted in this research work. Ethical approval for the use of animals in this study was granted by the Institutional Research Committee of the Mexican Social Security Institute (R‐2011‐1701‐3) and the Official Mexican Standard NOM‐062‐ZOO‐1999.

## Conflicts of Interest

The authors declare no conflicts of interest.

## Funding

This study is funded by Consejo Nacional de Ciencia y Tecnología México, 322334.

## Data Availability

The data that support the findings of this study are available on request from the corresponding author upon reasonable request.
